# Orbital Exenteration and Reconstruction Using a Free Radial Forearm Flap in Conjunctival Melanoma: Old but Gold

**DOI:** 10.7759/cureus.42572

**Published:** 2023-07-27

**Authors:** Ju Ann Tan, Ee Theng Khoo, Muath Mamdouh Mahmod Al-Chalabi, Hamidah Mohd Zainal, Wan Azman Wan Sulaiman

**Affiliations:** 1 Reconstructive Sciences Unit, Universiti Sains Malaysia (USM), Kota Bharu, MYS; 2 Plastic and Reconstructive Surgery, Hospital Kuala Lumpur, Kuala Lumpur, MYS

**Keywords:** radial forearm free flap, radial forearm flap, conjunctival melanoma, orbital reconstruction, orbital exenteration

## Abstract

Conjunctival melanoma is a rare and potentially deadly tumor. Therefore, adequate oncological resection is essential, commonly leading to total orbital exenteration, which causes patients' extensive functional and cosmetic impairment. As a result, it is essential to reconstruct the orbital region post-exenteration to obliterate the cavity, provide adequate and pliable cutaneous covering, and restore a stable vascularized tissue that can withstand adjuvant radiotherapy. In recent years, the techniques used for orbital reconstruction have included the transorbital temporoparietal fascial flap, the anterolateral thigh flap, and local flaps, such as the paramedian forehead flap. A free radial forearm flap is currently not commonly used for orbital reconstruction due to potential donor site morbidity and cosmetic issues. In our case, we report a free radial forearm fasciocutaneous flap that has been utilized with promising surgical outcomes to reconstruct the orbital region following orbital exenteration.

## Introduction

Malignant melanoma of the conjunctiva is an exceptionally rare and potentially life-threatening malignancy affecting the orbit, characterized by its aggressive nature. Conjunctival malignant melanoma accounts for a mere 2% of all orbital malignancies [1,2]. The reported incidences of conjunctival melanoma are less than one case per million population, with an annual age-adjusted incidence rate of just 0.15 in the Asian population [3,4]. This type of melanoma is often associated with primary acquired melanosis and nevi. Due to its rarity, well-established treatment protocols and comprehensive studies on overall survival rates are lacking.

In most orbital malignancies, orbital exenteration is often unavoidable to achieve local disease control. However, orbital exenteration is an extensive and disfiguring surgery with significant functional and psychological impacts on patients. The method of orbital reconstruction post-exenteration depends on factors, such as the extent of the surgical defect, the presence of preoperative radiotherapy, and any plans for future prosthetic implants.

In this case, we present a patient who underwent total orbital exenteration due to conjunctival malignant melanoma. Immediate orbital reconstruction was performed using a free radial forearm fasciocutaneous flap. We successfully reconstructed the orbital defect by providing the patient with good surgical outcomes. The free radial forearm flap's versatility and reliability make it a valuable head and neck reconstruction option.

## Case presentation

**Figure 1:**
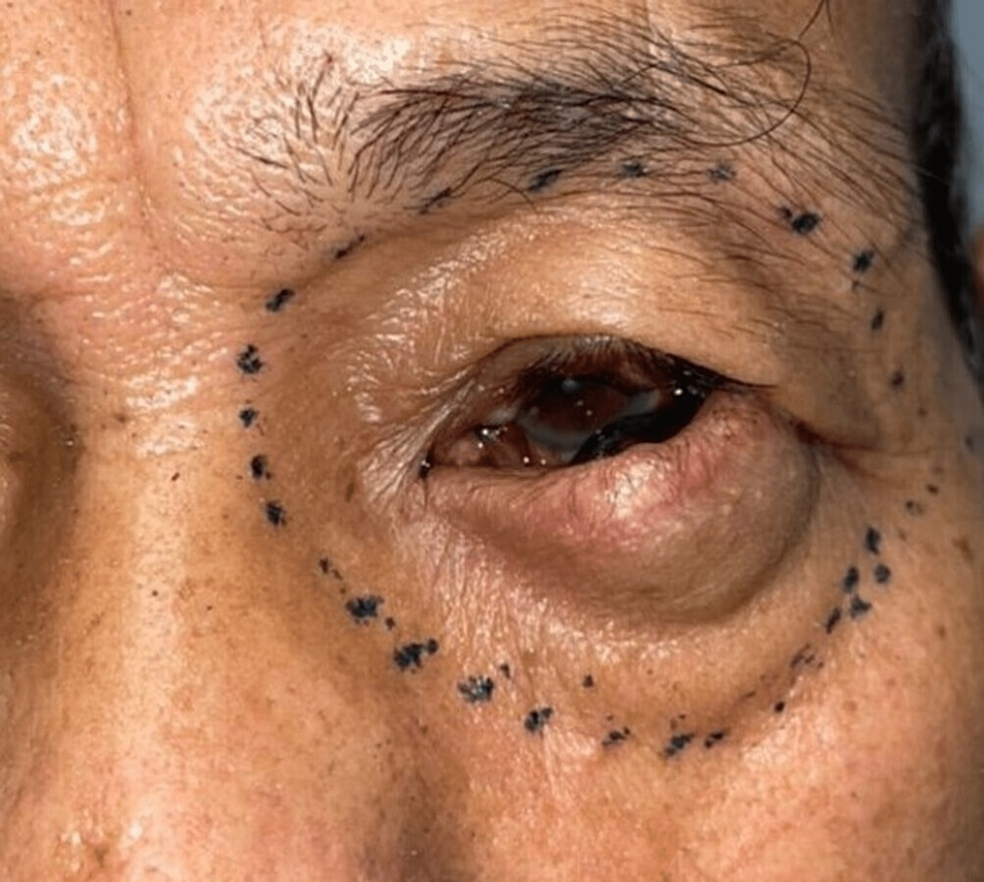
Swelling over the left lower eyelid with the appearance of left intraocular pigmentation.

A 74-year-old Malay gentleman, formerly a farmer, presented to an ophthalmologist with a six-month history of swelling over his left lower eyelid. It all began with the appearance of a pigmented lesion in that area, as shown in Figure 1.

He denied any loss of vision or blurring. A transconjunctival biopsy was performed on the tarsal-conjunctival lesion, and the histopathological report confirmed it as malignant melanoma. Further imaging through contrast-enhanced computed tomography (CECT) of the orbit revealed the presence of a heterogeneous mass arising in the tarsal conjunctiva adjacent to the globe but without infiltrating it. No extension into the extraconal or intraconal space was observed. Multiple subcentimeter intra- and extra-parotid lymph nodes and left neck lymphadenopathy suggested possible metastasis.

Oculoplastic; plastic surgery; oncology; ear, nose, and throat (ENT); and anesthesiology specialists conducted a multidisciplinary team discussion. After careful consideration and consultations with the patient and his family, a decision was made to proceed with left total orbital exenteration, right modified radical neck dissection, left total parotidectomy, tracheostomy, and soft tissue coverage using a free tissue transfer. The patient consented to the orbital exenteration without an orbital prosthesis.

**Figure 2:**
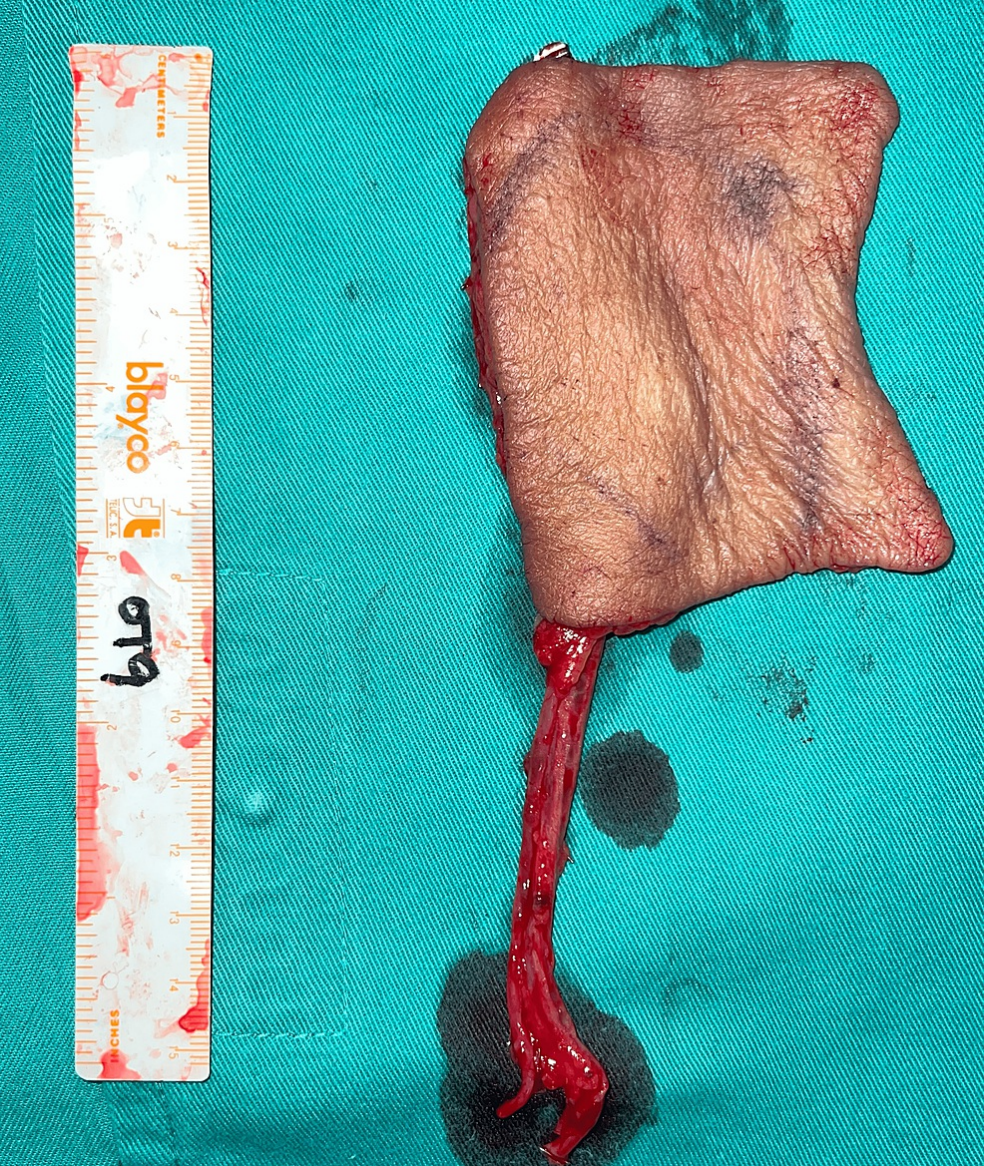
Free radial forearm fasciocutaneous flap along with a radial artery and vena comitans.

The operation was carried out sequentially. The ENT team performed the right modified radical neck dissection and total parotidectomy. The oculoplastic team conducted a wide local excision, including the excision of both the upper and lower eyelids, resulting in a defect measuring 6x5 cm post-exenteration of the right orbit. A left free radial forearm fasciocutaneous flap was employed for soft tissue coverage. Flap planning and marking were done preoperatively, and the Allen test was performed. A free radial forearm fasciocutaneous flap measuring 9x6 cm was harvested in the suprafascial plane, along with a radial artery and vena comitans, as shown in Figure 2.

The flap was meticulously folded and inset into the concavity. A pedicle was tunneled over the temporal region, and micro-anastomosis was performed using the superficial temporal artery and vein as recipient vessels. A split-thickness skin graft (SSG) harvested from the anterior thigh was applied as a sheet graft over the left forearm donor site.

**Figure 3:**
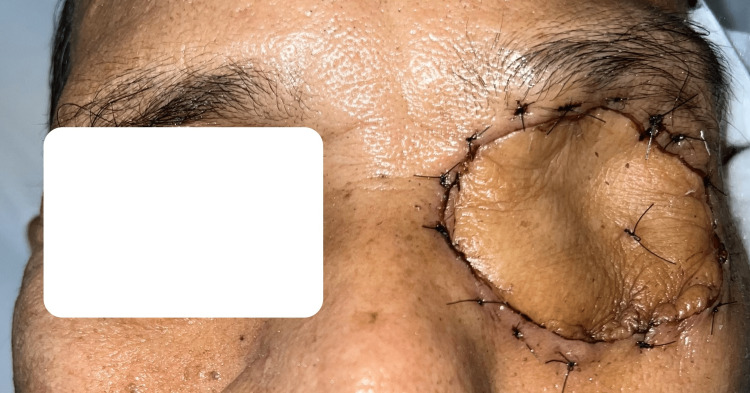
Appearance of the healthy survived free radial forearm flap covering left eye defect two weeks post-operatively.

Post-operatively, the flap healed well with no complications. The radial forearm flap over the orbital region was pliable and not bulky, as shown in Figure 3.

The left forearm donor site showed complete skin graft take with no contracture and no effect on the range of movements over the wrist joint. The patient reported having a normal grip function. He received intensive care for three days, could ambulate by day five, and was discharged on the 14th day after the operation, free of any post-operative complications. Subsequently, the patient was referred to the oncology department for adjuvant radiotherapy to complement his treatment.

## Discussion

Conjunctival malignant melanoma is a rare cancer characterized by thickened, hyperpigmented lesions. Notably, most of these melanomas (60-92%) are found in the bulbar conjunctiva, likely due to its direct exposure to sunlight [5]. A poorer prognosis is associated with palpebral, forniceal conjunctiva, plica semilunaris, and caruncle melanomas [6]. Lymphatic drainage is the main route of spread for conjunctival melanoma, with tumors in the nasal conjunctiva more likely to metastasize to submandibular nodes. By contrast, tumors in other conjunctival regions tend to metastasize to pre-auricular or cervical lymph nodes. Distant metastasis can occur through hematogenous spread to distant organs [7].

Given the aggressive nature of conjunctival melanoma and its high risk of relapse and metastasis, the treatment options remain challenging. The primary treatments include complete surgical resection through wide local excision, enucleation, or exenteration combined with cryotherapy. Adjuvant radiotherapy is employed to prevent recurrence and metastasis.

In cases where orbital exenteration is required, the reconstruction process can be complex and requires careful preoperative planning. The choice of reconstruction technique is influenced by several factors, such as the size of the defect, the need for postoperative adjuvant radiotherapy, and the patient's desire for prosthetic rehabilitation. Reconstruction aims to obliterate the defect, separate the nasal and orbital cavities, seal the skull base, and provide vascularized tissue to create a safe wound for adjuvant radiotherapy. The secondary goal is to achieve aesthetic function and optimize the defect for orbital prosthetics.

In cases of isolated orbit exenteration without bony resection, the main objective of reconstruction is to create a well-healed concavity for prosthetic placement. Total exenteration cases where the patient does not wish for a prosthesis require reconstruction using thin, pliable, and vascularized tissues to close the connection between the orbit and the intracranial compartment while achieving an aesthetically pleasing outcome. Options for such cases range from secondary granulation, SSG, and local flaps to free tissue transfer.

SSG is commonly employed when there is no involvement of the sinonasal or intracranial cavities. It offers faster healing time, minimizing delays in adjuvant therapy. However, there is a higher risk of postoperative infection and partial or complete skin graft loss, especially in patients requiring post-SSG radiotherapy. Therefore, SSG for cavity reconstruction is more suitable for patients needing a prosthesis who are not undergoing adjuvant radiotherapy. Some reports have highlighted the use of dermal matrices for orbital cavity reconstruction, reflecting advancements in surgical techniques [8].

Regional flaps harvested from the scalp, forehead, galea, and pericranium can be used for reconstruction. The temporoparietal fascia flap (TPFF) offers improved pliability and contour to the orbital cavity [9]. In cases where free flap reconstruction is not feasible, the paramedian forehead flap can be a reliable option for orbital reconstruction [10].

In situations requiring free tissue transfer for reconstruction, the need for bony reconstruction becomes a significant factor. Locally advanced and aggressive orbital malignancies may necessitate orbital exenteration with maxillectomy to ensure adequate surgical margins. In such cases, the primary goal of reconstruction is to eliminate any connections between the orbit and the intracranial and nasal cavities. An osteocutaneous scapular flap is a suitable option if a vascularized bone flap is needed.

In cases of orbital exenteration without bony resection, reconstruction of the orbital region using a musculocutaneous or fasciocutaneous flap can be a good option [11]. In recent years, there has been a decline in the use of the radial forearm flap for orbital reconstruction. This can be attributed to the advancement of surgical and microvascular techniques, which allows surgeons to explore other reconstructive options, such as perforator-based flaps. Introducing perforator flaps, such as the anterolateral thigh flap, deep inferior epigastric artery perforator flap, or scapular flap, has provided surgeons with viable alternatives for orbital reconstruction. Compared to the radial forearm flap, these flaps offer advantages, such as a larger surface area, improved aesthetics, and reduced donor site morbidity. One key concern in utilizing a free radial forearm flap in orbital reconstruction is the functional and cosmetic impairment over the donor site. Harvesting the radial forearm flap can result in a noticeable scarring area and contour deformity in the exposed area of the forearm. All these reasons have resulted in declining free radial forearm flap usage in recent years.

However, due to its numerous advantages, the radial forearm flap is a valuable option for head and neck reconstruction. It provides a large, reliable, and versatile source of well-vascularized tissue for reconstruction. The flap can be harvested with a long vascular pedicle, allowing for flexibility in positioning and shaping the tissue to reconstruct complex defects. The radial forearm fasciocutaneous flap can be harvested as a thin and pliable tissue, which allows better contouring and adaptation to the intricate anatomy of the orbital region. A radial forearm flap provides vascularized tissue to the wound bed, promotes better healing, reduces the risk of infection, and enhances the success of subsequent adjuvant therapies, such as radiotherapy.

Furthermore, the radial forearm flap has a low rate of complications and donor site morbidity. With recent modifications, suprafascial dissection of the radial forearm flap reduces donor site morbidity and a higher rate of complete skin graft take [12]. In addition, donor site morbidity does not significantly affect hand function, as the forearm's functional integrity remains primarily preserved. In our case, the patient is an elderly patient whose scarring over the forearm region did not impact his daily life, and his hand grip function and range of motion remained normal compared to pre-operatively. Thus, utilizing a free radial forearm flap in such a scenario provides a better surgical outcome, reduces the complexity of the operation, and reduces the operating time for elderly patients, leading to a better postoperative recovery.

Although there may be a decrease in the recent use of the radial forearm flap for orbital reconstruction, primarily due to the availability of alternative reconstructive options and evolving surgical techniques, they remain an important and valuable consideration for head and neck reconstruction.

## Conclusions

While the use of the radial forearm flap for orbital reconstruction has declined recently, they should still be considered a viable option in some instances, particularly in older patients, where the potential donor site morbidity may be less significant. Moreover, when minimal hand motion is affected, the advantages of the radial forearm flap can outweigh its drawbacks. The radial forearm flap continues to offer advantages in terms of versatility, ease of harvesting, and reliable vascular supply. Therefore, in some selected cases where the morbidity of the flap can be considered acceptable, it remains a valuable option for orbital reconstruction.
